# Neurobiology of opioid dependence in creating addiction vulnerability

**DOI:** 10.12688/f1000research.8369.1

**Published:** 2016-07-19

**Authors:** Christopher J. Evans, Catherine M. Cahill

**Affiliations:** 1 Hatos Center for Neuropharmacology, Semel Institute for Neuroscience and Human Behavior, University of California, Los Angeles, CA, 90095, USA; 2 Departments of Anesthesiology & Perioperative Care and Pharmacology, University of California, Irvine, CA, 90095, USA; 3 Department of Biomedical and Molecular Sciences, Queen's University, Kingston, Ontario, K7L 3N6, Canada

**Keywords:** Learned Associative Model, pass-forward allostasis, withdrawal relief, aversive states, opioid epidemic

## Abstract

Opioid drugs are potent modulators of many physiological and psychological processes. When given acutely, they can elicit the signature responses of euphoria and analgesia that societies have coveted for centuries. Repeated, or chronic, use of opioids induces adaptive or allostatic changes that modify neuronal circuitry and create an altered normality — the “drug-dependent” state. This state, at least that exhibited by those maintained continuously on long-acting opioid drugs such as methadone or buprenorphine, is generally indistinguishable from the drug-naïve state for most overt behaviors. The consequences of the allostatic changes (cellular, circuit, and system adaptations) that accompany the drug-dependent state are revealed during drug withdrawal. Drug cessation triggers a temporally orchestrated allostatic re-establishment of neuronal systems, which is manifested as opposing physiological and psychological effects to those exhibited by acute drug intoxication. Some withdrawal symptoms, such as physical symptoms (sweating, shaking, and diarrhea) resolve within days, whilst others, such as dysphoria, insomnia, and anxiety, can linger for months, and some adaptations, such as learned associations, may be established for life. We will briefly discuss the cellular mechanisms and neural circuitry that contribute to the opioid drug-dependent state, inferring an emerging role for neuroinflammation. We will argue that opioid addictive behaviors result from a learned relationship between opioids and relief from an existing or withdrawal-induced anxiogenic and/or dysphoric state. Furthermore, a future stressful life event can recall the memory that opioid drugs alleviate negative affect (despair, sadness, and anxiety) and thereby precipitate craving, resulting in relapse. A learned association of relief of aversive states would fuel drug craving in vulnerable people living in an increasingly stressful society. We suggest that this route to addiction is contributive to the current opioid epidemic in the USA.

## Introduction

An opioid epidemic has emerged over the past decade in the United States causing overdose deaths fast approaching numbers dying from car accidents, with about 17,000 annual deaths from opioid therapeutics and 8200 deaths from heroin overdose in 2014 ^1, 2^. Sales of prescription medications have skyrocketed, with an increase in retail sales of 1293% for methadone, 866% for oxycodone, and 525% for fentanyl from 1997–2007 ^3, 4^. Similarly, the increase in therapeutic opioid use in the United States in milligrams per person increased 402% from 1997 to 2007. Although reformulation of oxycodone to an abuse-deterrent form has helped curb the oxycodone epidemic, the migration to other opioids following drug dependence remains problematic, especially the migration to heroin ^3, 4^. The statistics are disturbing, and in 2011, the White House released an action plan entitled “Epidemic: Responding to America’s Prescription Drug Abuse Crisis”. Although opioid therapeutic prescriptions for pain are largely blamed for creating the epidemic, other factors undoubtedly contribute to the problem, including the low price of heroin and environmental stressors leading to anxiety and depressive states. The conundrum of effective treatment of pain while minimizing opioid prescription abuse is a major political challenge that remains unresolved.

Drug dependence is defined as a drug-induced state in which, upon cessation of the drug, physical and/or psychological withdrawal symptoms occur. Like many definitions, this is a fuzzy one. Though not considered dependence, even a single drug exposure can cause an acute withdrawal syndrome as the body and psyche re-equilibrate. For example, hangovers from alcohol or the crash after a night of psychostimulants are (at least in part) the result of the nervous system readjusting from a drug-induced state. A commonly held, and mistaken, view of opioid dependence is that it is entirely synonymous with adaptive states that elicit the autonomic physical symptoms of withdrawal (such as lacrimation, runny nose, hot/cold sweats, cutis anserine, or diarrhea). However, physical withdrawal is just one component of opioid abstinence syndrome, and the anxiogenic and negative affective states that evolve following abstinence ^5, 6^ are likely more salient for driving addictive behaviors ^7^. Drug dependence is not a unique phenomenon to opioids, as psychostimulants and other drugs of abuse can elicit an abstinence syndrome. Like opioids, psychostimulant abstinence precipitates dysphoria ^8^, emotional withdrawal, and hypo-dopaminergic tone ^9^, but opioids, the focus of this review, may trigger different vulnerabilities for addiction and relapse.

Drug cessation in the opioid-dependent state invariably elicits the opposite physiological and psychological manifestations from the acute drug state, resulting in physical withdrawal and the genesis of negative affect. Thus, opioid drugs such as morphine or oxycodone when given acutely induce euphoria and a sense of well-being. Opioids also reduce stress hormone secretion ^10, 11^ and are known to be effective anxiolytics and anti-depressants when given acutely ^12, 13^. Indeed, buprenorphine reduces depression severity scores in patients with treatment-resistant depression ^14, 15^. In contrast to the acute drug effects, drug cessation from the opioid-dependent state induces agitation/panic attacks and dysphoria. Some drug-dependent people can tolerate the autonomic, hyperalgesic, anxiogenic, and/or affective components of withdrawal, whilst other individuals find it an almost insurmountable barrier, dreading withdrawal or having uncontrollable craving for relief from the withdrawal state (see discussion below). There are individual differences both in the initial propensity to take drugs and in the propensity to develop addictive behaviors ^16^. Following drug cessation, some individuals may never develop drug cravings, whilst others may retain a strong propensity to seek the drug state and exhibit addictive behaviors, especially following drug-associated triggers or cues. For example, many patients on opioid pain medications, though very much drug dependent, will go through withdrawal on drug cessation but never display addictive behaviors that are detrimental to their well-being. This is possibly due to pain patients having a learned association with pain relief (negative reinforcement). However, chronic pain patients with high catastrophizing scores are at risk of opioid misuse, as catastrophizing is associated with craving in patients prescribed opioid analgesics (a strong determinant for opioid misuse) ^17^. Similarly, impulsivity facets ^18^ and distress intolerance ^19^ (the perceived or actual inability to manage negative emotional and somatic states) were also identified to be a risk factor of prescription opioid misuse in the context of chronic pain treatment. Though tolerance and withdrawal symptoms have been retained in the revised Diagnostic and Statistical Manual of Mental Disorders, 5th Edition (DSM V) definition for substance use disorder, they are not stand-alone diagnostic criteria for this disorder.

So what are the underpinnings of the behavioral disease state of opioid addiction? One addiction theory that has been proposed stems from many decades of addiction research, including that of Robbins, Everitt, Wise, Berridge, Kalivas, Robinson, and Piazza ^20^. This theory posits that a reward learning process occurs as the first step to addiction, followed by a second step of escalated drug use in vulnerable individuals with hypo-dopaminergic systems and impaired prefrontal cortex inhibitory control. The third step, leading to the addiction phenotype, is postulated as a result of allostatic drug-induced states in reward circuitry leading to a strong desire for drugs (incentive sensitization) ^21– 23^. In this model, positive reinforcement is emphasized and the driver for addiction is the modified reward circuitry and loss of inhibitory control, with inference of altered synaptic plasticity in cortical striatal circuitry ^23^ and switching from goal-directed to habit circuitry ^24^. The formative studies for this theory are predominantly derived from psychostimulant self-administration studies and emphasize individual vulnerabilities. In what is largely considered a contrary theory, the allostatic load and “antireward” that is created in the drug-dependent state as a key driver of addiction has been proposed by Koob and colleagues ^7, 25, 26^. The importance of drug withdrawal and aversive states as an addiction driver is argued in a commentary addressing the general theory by Piazza and Deroche-Gamonet ^20^. In this “dark side” model, continued drug taking leads to a negative emotional state upon drug cessation that drives negative reinforcement, involving the brain reward and stress systems ^27^. The increased incentive value of opioids during withdrawal can be learned and mediates enhanced drug seeking during subsequent withdrawal from the drug ^28^. For reasons noted later in this review, the dark side of drug taking has a particularly strong rationale for opioid addiction.

The drive for relief from the withdrawal state (negative reinforcement) would provide motivation for continued opioid drug taking and is consistent with the often-reported rationale for opioid addicts to continue drug taking to “avoid withdrawal”. In addition to the physical withdrawal, there is also a protracted abstinence syndrome characterized by negative affect that also drives drug craving that may last much longer. However, drug craving as a direct consequence of negative reinforcement to alleviate psychological withdrawal does not account for why addictive behaviors and craving manifest sometimes months or years after drug cessation when, presumably, withdrawal has terminated. Further, this theory does not account for incubation of drug craving. Why does individual susceptibility for addictive behaviors exist if all individuals experimenting with opioid drugs do not transition to addiction? Models that describe a modification of direct negative reinforcement, whereby withdrawal-based learning ^28^ and avoidance of negative affect are the major drivers of addictive behaviors, resonate well with the mechanisms that we propose are particularly relevant to opioid addiction ^25, 29– 31^. There is also evidence that opioids increase the risk of depression recurrence even after controlling for pain, psychiatric disorders, and opioid misuse ^32^. These data suggest that patients with remitted depression who are exposed to opioid analgesics were more likely to experience a recurrence of depression than those who remained opioid free, which may contribute to future opioid use or misuse to alleviate depressive symptoms.

**Figure 1.**
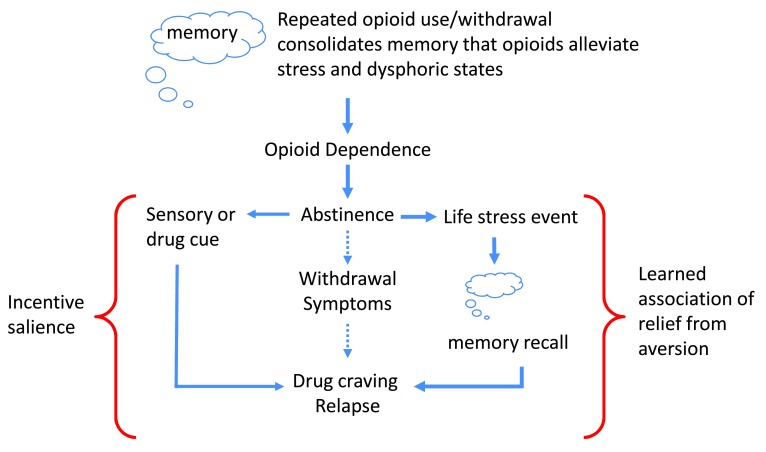
Learned association of relief of aversive states. Initial opioid use is reinforced by euphoria and positive mood, promoting further drug use. However, the motivation for opioid taking changes with repeated use, where positive reinforcing effects of the drug wane in comparison to the drive to alleviate withdrawal effects (negative reinforcement). With repeated drug use, opioid dependence develops and the learned association with relief of the aversive withdrawal state is reinforced. Following abstinence, the risk of relapse can be driven by three paths. The first is by direct negative reinforcement and relief of withdrawal. The second path would be sensory or drug cues (e.g., drug paraphernalia, familiarity of location to previous drug use, scent, etc.) and drug access (left side of figure), where incentive salience drives craving and loss of inhibitory control drive relapse. The other (right side of figure) is the trigger of life stress events that recall the memory of learned association between drug taking and aversion relief. In individuals with pre-existing negative affective states, the prediction is that the initial opioid use would immediately be associated with negative reinforcement in alleviating dysphoric symptoms and a) memory consolidation would be established more rapidly and b) the opioid would have increased salience during withdrawal for creating associative memories due to exacerbated dysphoria.

In this review, we consider the psychological adaptations revealing anxiety and negative affective states during opioid withdrawal as a disease primer, analogous to a wound for a systemic bacterial infection. We will consider opioid addiction as an associative learning disorder, with parallels to post traumatic stress disorder (PTSD). We will argue that it is not necessarily positive reinforcement or the negative reinforcement to avoid withdrawal that drives opioid addiction, but it is a learned association of drug relief from an aversive mental state, either pre-existing or created by withdrawal, that drives craving and the resultant addictive-like behaviors in susceptible individuals ( Figure 1).

## Learned association of relief from aversion

So how might the disease of addiction develop and what are the susceptibilities? One parsimonious explanation is that learned associations are driving drug craving, and once the associations are established they take a paramount role in driving aberrant behaviors. Analogous to the epidemiology of addiction, where many individuals are exposed to drugs but only a subset become addicted, many individuals are exposed to traumatic events, yet few will go on to develop PTSD. In PTSD, the aberrant behavioral responses are avoidance and hyper-vigilance to evade cues of an established fear memory, whereas in opioid addiction a response to evade aversive states would simply be opioid drug taking. Both disorders are characterized by intrusive thinking, which is a shared endophenotype of many neuropsychiatric disorders ^33^. Interestingly, there is a very high co-morbidity of PTSD and depressant drug abuse (opioids, alcohol, and benzodiazepines), and recent research has strongly implicated opioids as being protective against the acquisition of fear memories in PTSD ^34, 35^. Indeed, a commonality in etiologies between opioid addiction and PTSD may engage fear memory circuitry. It was recently suggested that while many factors likely contribute to the disproportionate co-occurrence of PTSD and substance abuse, one such factor may be a core psychological trait that biases some individuals to attribute excessive motivational salience to predictive cues, regardless of the emotional valence of those cues ^36^. An insightful recent review by Bali and colleagues ^37^ details the role of both endogenous and exogenous activation of the mu opioid system in the modulation of stress, dysphoria, and PTSD.

**Figure 2.**
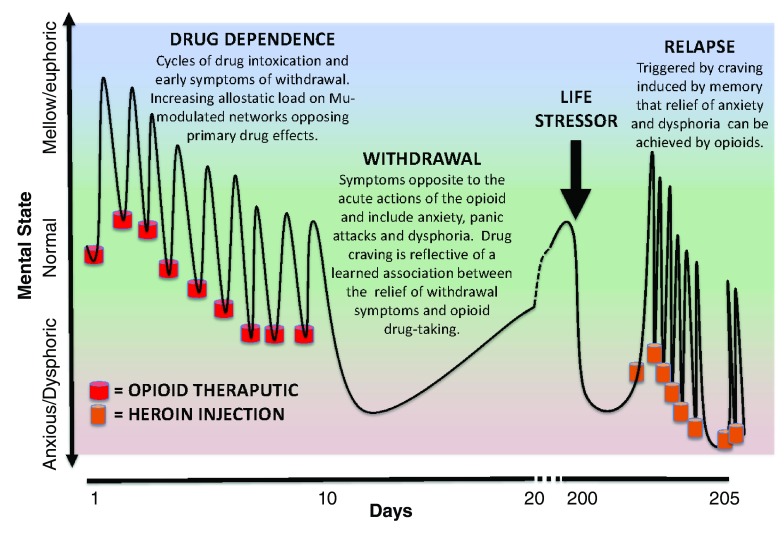
Laura’s pathway to heroin addiction. Here we provide a hypothetical scenario of how learned association of relief of aversive states could lead to the development of addiction and the key role of opioid dependence. Let us consider a disappointed teenager (Laura) who sprained her ankle during tryouts for the cheerleading team and was rejected, not making the squad. Knowing that opioids will alleviate the pain caused by the sprained ankle, Laura takes an opioid analgesic pill she knows is in the bathroom medicine cabinet, left over from her older brother’s prescription for a wisdom tooth extraction. The opioid pill takes away Laura’s pain from the sprained ankle and makes her feel relaxed (mellow and with elevated mood). She is less bothered about the tryout rejection while under the influence of the opioid. The next day, Laura decides to take another pill as the drug effects have worn off and both the ankle (physical) pain and the (psychological) pain of rejection have returned and are just as bad as the day before. The taking of the opioid medication again relieves the physical pain and takes her mind off her failure. Over the next 10 days, the drug taking cycle continues, but each day the pain-relieving effects are less (development of tolerance) and she develops a modest anxiogenic state prior to taking the drug each day (early signs of withdrawal). As the pain from the ankle sprain subsides, the association of drug taking becomes the relief of the anxious and dysphoric states that emerge as the drug wears off. On day 10, the supply of drugs from the medicine cabinet is exhausted and withdrawal sets in. Drug seeking would be a consequence during this withdrawal phase given the learned association that the aversive symptoms she is experiencing could be effectively relieved by taking the opioid (negative reinforcement). Although unlikely, given the ready access to illicit opioids throughout society, let’s assume that at this point in Laura’s life, she doesn’t actively seek out prescription or other opioids. The teen goes through physical withdrawal (over days) with protracted mood disturbances accompanied by drug craving that is triggered by memories that she could relieve dysphoric states by taking opioids. She doesn’t continue to seek opioids at this stage because the pain of rejection has dissipated and she is now engaged with other activities in her peer group. Over a year later, her boyfriend breaks up with her, her grades are not stellar, and she may not get accepted to the colleges she wants to go to. Laura becomes extremely stressed and feels unable to cope. The emergence of these negative symptoms initiates a craving for opioids that is triggered by learned associative memories. At this point in her life, she has the resources to access heroin and relapses. Laura’s relapse reinforces the associative memories that aversive states are relieved by opioids and the foundation for further addictive behaviors is strengthened. In this example, drug dependence and withdrawal contributes to the addiction vulnerability and the learned associative memories are of the negative reinforcement provided by opioid relief of anxiogenic and dysphoric states, and are triggered by a different stressor.

The ability of opioids to relieve the anguish of withdrawal (negative reinforcement) would create a clear internal message and association that “opioid drugs alleviate aversive states”. This association would be reinforced by repeated cycles of opioid intoxication and the inescapable anxiogenic and dysphoric withdrawal symptoms that follow each cycle ( Figure 2). It is not difficult to envisage that after time a learned association of opioid drug taking with the relief of dysphoric states becomes engraved into brain motivational memory circuitry. Thus, when the addict experiences a future life event (maybe after years of abstinence) inducing negative affect (e.g., sadness, disappointment, failure, and apathy), the learned association of drug taking with relief of similar symptoms (albeit created by drug withdrawal) will trigger drug craving and relapse ( Figure 1). In order for a drug to gain motivational salience in a specific state, it is presumed that its reinforcing effects must be experienced in that same state. We would argue that the ability of many different stressors (e.g., yohimbine or foot shock) to induce drug relapse following extinction ^38^ is an example of generalization of stress relief with drug relief of withdrawal-induced dysphoric states.

A learned relief of aversive states driving addictive behaviors fits well with many observations in addiction medicine and epidemiology. It would be expected that those susceptible to depression and anxiety would have increased vulnerability to opioid addiction, a relationship firmly established over many years of opioid research and with evidence for self-medication, shared vulnerability, and precipitation of disorders following opioid abuse ^39, 40^. Importantly, many stressors creating dysphoric states are unequivocally strong triggers of opioid and other drug relapse in humans, as well as in animal models ^41^. Furthermore, highly addictive opioid drugs such as heroin and fentanyl, which have a fast onset and short half-life, create a high frequency of vacillating states of intoxication and withdrawal, providing more learning trials to solidify associations ( Figure 2). Support for opioid craving triggered by dysphoric states is provided by various clinical studies. Electronic diaries of heroin and cocaine polysubstance abusers on methadone maintenance captured subject descriptors immediately prior to drug relapse ^42^. In this study, cocaine use was robustly associated with subjects reporting that they “saw drug”, were “tempted to use out of the blue”, “wanted to see what would happen”, and were in “good mood”. In contrast, those who reported heroin craving showed robust association with “feeling sad” or “feeling angry”, suggesting that expression of aversive states triggered opioid craving. Similarly, heroin use in opioid-dependent patients produced a decrease in anxiety that correlated with reduced levels of the stress hormone adrenocorticotropic hormone (ACTH) ^43^. Methadone or buprenorphine maintenance would allow the brain to exist in an opioid-dependent state without vacillation between drug intoxication and withdrawal states, and its success for addiction treatment may be in part due to extinction of established associations of relief of negative affective states by the drug. Fear of withdrawal is one of the primary reasons for continuation of treatment in buprenorphine- or methadone-maintained patients. For example, the majority (65–90%) of patients maintained on buprenorphine or methadone indicated “‘concern of withdrawal discomfort”’ as the primary reason for continuing therapy ^44, 45^. The fear of withdrawal was identified to cover a spectrum from mild anxiety to morbid, almost pathological fear ^46^. The majority of patient descriptors indicated buprenorphine-maintained therapy made them feel “normal”, “level-headed”, and “okay” ^45^. Interestingly, the finding that alpha-2 adrenergic agonists selectively block stress-induced reinstatement of heroin seeking in rodent models of addiction ^47, 48^ was recently shown to have translational efficacy. Thus, clonidine maintenance prolonged opioid abstinence, where the positive effect of clonidine was correlated with stress relief ^49^. Similarly, opioid addicts on naltrexone maintenance who received guanfacine (an alpha-2 agonist) decreased self-reports of stress and craving ^50^. High motivation for relief of aversive states is a key assumption and would indicate that individual responses to anxiety- or fear-provoking stimuli are likely to correlate with addiction susceptibility. This idea is supported by data showing that animals who attribute high levels of motivational salience to predictive cues, regardless of emotional valence, are susceptible to contextual fear ^51^. This latter study predicts that motivational salience to predictive cues may predispose individuals to vulnerability for acquiring psychiatric disorders, including PTSD and substance abuse ^51^. In PTSD, where fear states are poorly regulated and tolerated, there is high co-morbidity with opioid addiction ^52^.

An association with relief of negative symptoms would also predict that as society increases its levels of stress and depression, the opioid epidemic is likely to worsen due to more triggers for relapse. There are several indicators both in the past and in the present that environmental stressors are triggers for opioid addictive behaviors. While enlisted in the Vietnam War, many army men deployed overseas tried heroin (~35%). Of those who tried heroin, over 50% became addicted to the opioid ^53^. However, on leaving the high-stress environment and returning to their homes in the United States, only a very small percentage of the ‘addicted’ soldiers retained their heroin addiction. Importantly, the cohort of Vietnam combat veterans with PTSD who became addicted to sedative drugs (alcohol, marijuana, heroin, and benzodiazepines) retained their addiction upon returning to the United States and civilian life. Their drug use paralleled their PTSD symptoms and drug use was reported to mitigate symptoms ^54^. Hence, unlike the cohort described above, the continued stressor of PTSD was sufficient to preserve the addiction. Alternatively, these individuals may have a pre-existing neurobiological basis making them susceptible to both disorders ^51^.

Relevant to the current opioid epidemic, an article assessing 2013 data shows a recent increase in mortality in midlife White Non-Hispanic Americans (despite medical advances), whereas Black and Hispanic ethic groups had declining mortality ^55^. The increased mortality is fully accounted for by the increased overdose from drugs, alcohol abuse, suicides, and related external causes. Opioid overdose is highly implicated and indeed a disproportionally higher (30%) number of opioid prescriptions are being dispensed to White compared to Black families ^55^ and therefore there is higher accessibility of opioids for misuse in these families. Other factors have been proposed to account for these data ^56^, but it appears that there is a considerable underestimation of deaths due to opioid and benzodiazepine overdose ^57^. These findings are attributed to an increase in midlife stress of White males, and we propose that a learned association of opioid drug taking to alleviate psychological aversive states is a contributing factor for the increased mortality.

## Circuitry involved in addiction and withdrawal

The brain circuitry contributing to addiction has been studied for many years with distinct and overlapping networks implicated in drug liking, binge/intoxication, withdrawal, and drug wanting/craving ^21, 30, 58^. Predominant models have focused on the positive reinforcement that engages the mesolimbic reward circuitry, firstly goal-directed circuitry transitioning to the habit learning processes. Relevant to this review is the neurocircuitry that may be responsible for learning associations of drug-induced relief of aversive states ^59^. There are multiple brain stress systems and transmitters that have been identified to contribute to the drug-induced dysphoric and negative affective states, including corticotropin-releasing factor, dynorphin, vasopressin, hypocretin, etc. ^30^. Recently, the paraventricular nucleus of the thalamus (PVT)’s connectivity with the nucleus accumbens (an area of the striatum associated with both desire and dread ^21^) has been identified as a prominent circuit in relaying the aversion and creation of opioid withdrawal memory in a place-aversion assay ^60^ (albeit the PVT also contributes to appetitive learning ^61^). Thus, optogenetic silencing of this pathway suppressed both naloxone-precipitated physical withdrawal and naloxone place aversion in the dependent state. The implication from additional experiments focusing on the nucleus accumbens was that plasticity in dopamine receptor 2-expressing medium spiny neurons is a necessary circuitry component for the aversive learning of withdrawal. The PVT is implicated in stress reactivity, and its extensive input by all monoaminergic processes and the prelimbic prefrontal cortex—with outputs to the extended amygdala, cingulate cortex, and nucleus accumbens ^62, 63^—provides an opportunity to orchestrate or, at the very least, participate in aversive learning. Indeed, orexin neurons are involved in conditioned place aversion to morphine withdrawal ^64^, and orexin neurons innervating the PVT are involved in the physiological and behavioral response to stress ^65^. Upstream and downstream circuitry is currently unknown. A prominent norepinephrine input to the bed nucleus of the stria terminalis (BNST) from the ventral noradrenergic bundle contributes to anxiety associated with protracted opioid withdrawal. In contrast, the lesion of the locus coeruleus does not alter opioid physical withdrawal symptoms ^66, 67^.

Other brain areas are likely involved in memory and associative processes concerning withdrawal, which translate into circuitry driving craving. Learning circuitry that is “goal directed” occurs in the dorsal medial striatum and frontal/orbitofrontal cortical striatal circuits. On repeated learning trials, transition to dorsal striatum and sensorimotor cortical circuits occurs for “habit learning”, and disruption of the balance between goal-directed and habit circuitry has been proposed for compulsive habitual responses of addicts ^68, 69^. An area of the brain that is involved in emotional learning and is highly active in the craving of many drugs of abuse is the amygdala ^30, 70^. Circuitry activating ERK and CREB signaling in the central amygdala has been strongly implicated in incubation of opioid craving ^71^. Furthermore, the basolateral amygdala was shown to be involved in the retrieval of opioid cue memories and was proposed as a site of intervention with a reconsolidation strategy ^72^. Furthermore, lesions of the basolateral amygdala prevented conditioning responses to naloxone that suppressed food reward seeking during sustained opioid treatment ^59^. We propose that the relevant circuits driving the learning for relief of aversion to elicit craving are likely to be created in circuitry orchestrated by the PVT and BNST and that modifications in learning circuitry modulate the basal lateral and central amygdala to drive craving.

## Cellular mechanisms and pass-forward allostasis

**Figure 3.**
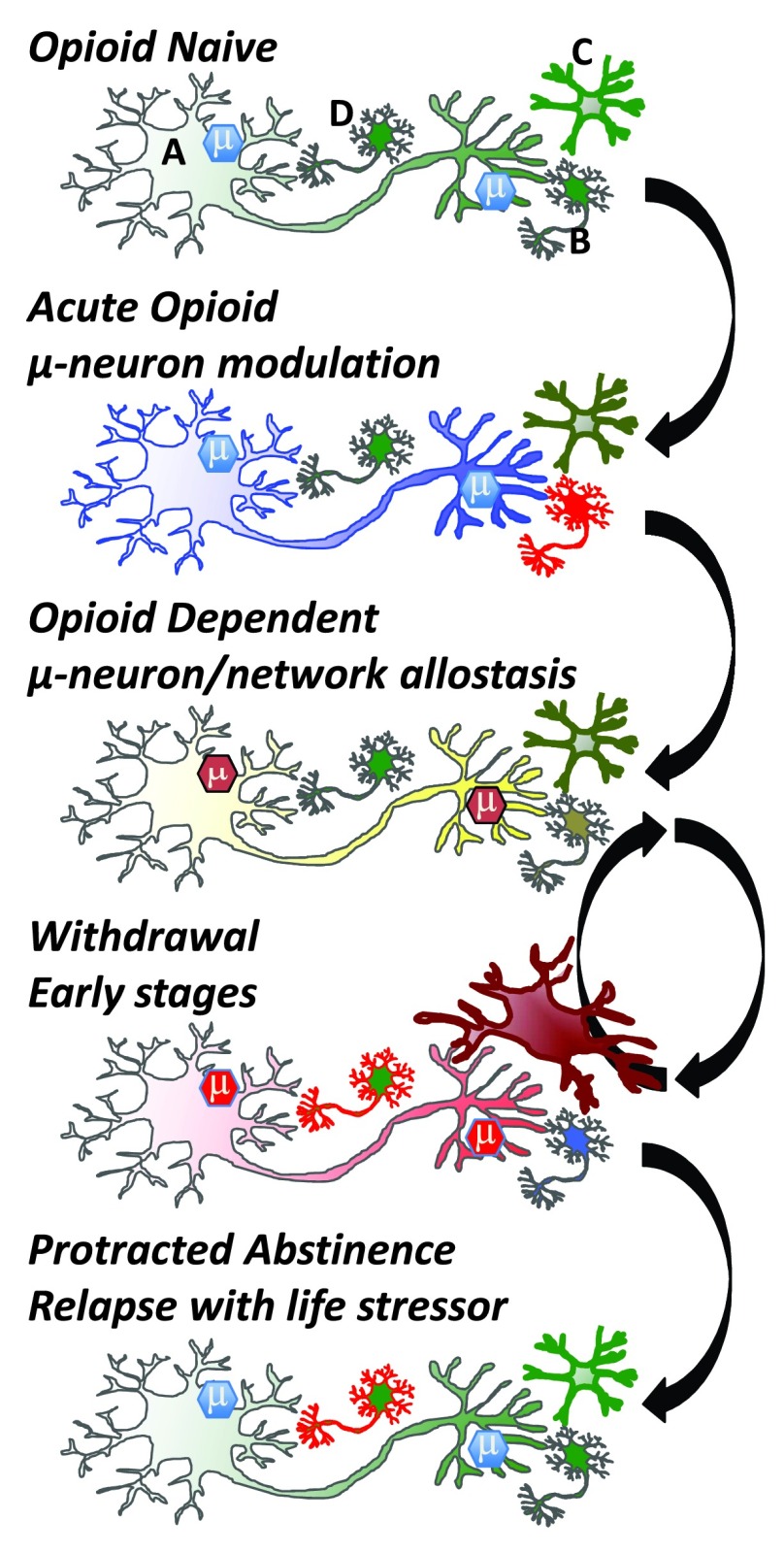
Cellular adaptations to opioids. In the ‘Opioid Naïve’ state, mu opioid receptor expressing cells (e.g., GABAergic neuron; top panel, neuron A) modulate reward circuitry and many other neurons (top panel, neuron B) in the brain and periphery. Brain microglia (top panel, cell C) are normally in a quiescent surveillance state. Acute opioid administration activates mu opioid receptors, which couple to inhibitory G proteins and generate an active mu-signalosome (μ) that inhibits cell activity and neurotransmitter release. In the brain, mu opioid receptors are often on GABAergic inhibitory neurons and thus can activate adjacent neurons (B) via disinhibition. Microglia may also be activated directly by opioid drugs, although this is debated. In the ‘Opioid Dependent’ state, opioid receptor expressing neurons adapt to the continued presence of drug. Modifications occur in the µ-signalosome, neuronal proteome, and transcriptome, alongside modifications to neuronal morphology (e.g., spine density and dendritic arborization). Maintained activation of mu-opioid receptors begins a process of cellular, network, and system adaptations. On drug cessation withdrawal is triggered, and the adaptive (allostatic) changes that occur in the drug-dependent state rebound in a temporally (hours to years) orchestrated resetting of neurons and networks. Withdrawal symptoms are opposing the acute actions of opioids whereby neurons inhibited by opioid activation become excited during withdrawal. Other cells are engaged during withdrawal, including microglia (Cell C) and neurons within the anxiogenic learning circuitry (Cell D). Eventually, weeks to months after drug cessation, many networks re-establish a close-to-normal state, but neurons encoding memory circuits of withdrawal (cell D) and associated memories that drugs relieve aversive states can be triggered by stressful or aversive life events following ‘Protracted Abstinence’. Green: resting state, blue: inhibited state, yellow: near-normal dependent state, red: activated state. Arrows denote transition between states.

Driving the behavioral transformation of cellular phenotypes leading to an opioid-dependent state and tolerance are a plethora of adaptive responses, beginning at the opioid receptor itself, which modulates multiple signaling pathways that adapt to continued presence of the opioid drug ( Figure 3). Thus, opioid receptors desensitize and down-regulate, which are processes mediated by kinases and receptor-associated proteins such as arrestin isoforms modulating the mu receptor signalosome and receptor trafficking ^73^. Furthermore, curtailing mu opioid receptor signaling occurs downstream of the opioid receptors. Adenylate cyclase supersensitivity upregulates the adenylate-cyclase signaling system and opposes the inhibitory signaling effects of opioid receptors ^74^. The neuron adapts in many ways (e.g., transcriptional and dendritic spine changes) to the continued presence of the drug and reaches a new allostatic state—the drug-dependent state ( Figure 3). These adaptive processes clearly occur in neurons containing opioid receptors but will have a ripple effect on circuits and a pass-forward allostasis, first in local networks and then in system networks that can extend far from the initial site of opioid action ( Figure 3). It is likely that nearly every neuron in the brain is touched by pass-forward allostatic changes in response to opioids given the extensive behavioral repertoire elicited by opioid drugs and the broad neuronal distribution of mu opioid receptors ^58^. With feed-forward allostasis likely occurring in most neurons of the brain following opioid dependence, circuitry perturbation will be extensive. Such processes will create an allostatic load that, following drug cessation, will initiate cellular and network activities opposite to the initial acute drug action engaging the BNST, PVT, and amygdala circuitry.

In addition to neuron-mediated adaptations, other adaptive processes also contribute to the opioid-dependent state. Microglia (the resident immune cell in the central nervous system) engulf dying cells, are responsible for surveillance of non-host pathogens of infectious agents, and play a critical role in neuronal guidance during development. During surveillance mode, in a healthy brain, microglia are ramified with long processes constantly surveying the environment of the brain parenchyma. However, once activated, microglia can cause remodeling of circuitry due to release of various factors including cytokines, chemokines, and growth factors such as brain-derived neurotrophic factor (BDNF). Microglia activated in neurodegenerative diseases are known to prune dendritic spines, which may contribute to the cognitive impairment and learning deficits in such diseases. Opioid drugs will activate microglia in various brain regions, including the limbic circuitry, which is responsible for emotion and reward ^75– 78^, although the mechanism underlying this activation remains debated and is not inherently dependent on Toll-like receptors, as previously suggested ^78, 79^. Similar to injury or pathogenic activation, opioid-induced activation causes microglia to release various factors, which in turn contribute to opioid tolerance and dependence, as well as the paradoxical opioid-induced pain (hyperalgesia) ^80^. Opioid dependence initiates microglial activation in the ventral tegmental area that causes a blunting of mesolimbic reward circuitry via BDNF and altered chloride homeostasis in GABAergic neurons ^76^. Other behavioral consequences of this microglial activation, such as perturbing affect and motivation, or their contribution to allostasis and adaptations that lead to opioid dependence and addiction remain elusive. One of the important aspects of microglial activation that has not been addressed is whether repeated withdrawal may precipitate the neuroimmune response rather than the opioid drugs themselves. The genesis of an opioid-dependent state and tolerance is typically with intermittent dosing that, by design, causes repeated withdrawal symptoms. It is not known if microglial activation will occur with sustained-release formulations or pump delivery that would prevent withdrawal. It is tempting to speculate that microglia may be critical for the neuroadaptations responsible for the learning component of associating relapse of drug use with withdrawal relief, including the alleviation of anxiety or negative affect ( Figure 3). Interestingly, a microglial inhibitor was reported to improve motivation without altering pain sensation in chronic pain patients ^81^, suggesting that microglia contribute to negative affect. Since microglia are critical for axonal guidance and regulate spine density, they have the potential for re-shaping the nervous system to produce the adaptations and allostasis that create new memories and associations of drug use with alleviation of withdrawal.

## Concluding remarks

The ‘brain disease model of addiction’ has been challenged and rebutted in recent articles, along with the use of animal models for addiction-related research ^82– 85^. In this review, we advocate an addiction disease model whereby a pre-existing disorder (such as depression or anxiety) or withdrawal from opioids creates associative memories that opioid drugs can provide relief. The association of the relief of these aversive states is generalized to other aversive states and creates drug craving that is precipitated by a subsequent stressful life event or drug cue. We suggest that opioid addiction may be qualitatively similar to other depressant drugs (alcohol and benzodiazepines) but may be different than mechanisms underlying psychostimulant addiction. Considering addiction as driven by a learned association of withdrawal relief has several implications. It would favor partial agonist treatments with a long half-life so as to avoid cycles of learning during intoxication and withdrawal phases. Buprenorphine has obvious benefits, given it is a weak partial agonist with a long-half life and kappa opioid antagonist activity blocking stress-induced opioid relapse ^86^. Other potential treatments may consider attenuating neuroinflammation, as neuronal-microglial communication may both contribute to negative affect and consolidate memories given the importance of microglia in neuronal pruning. Finally, the effective treatment of anxiogenic and dysphoric states would be critical to avoid relapse, and strategies could be considered to disrupt memory processes associating drug taking with relief of aversive states (perhaps reconsolidation).
